# Supplementary material for: Validation of enhanced kinect sensor based motion capturing for gait assessment
Source: PLoS One. 2017 Apr 14;12(4):e0175813. doi: 10.1371/journal.pone.0175813 (PMC5391956; doi:10.1371/journal.pone.0175813)
Supplement: S1 Appendix — (PDF) [file pone.0175813.s001.pdf]

Calculations for spatial calibration

We have modeled the problem of finding a transformation from any sensor coordinate frame to the coordinate frame of the first marker as path finding problem in a bipartite undirected graph $G = (V, E)$ with $V = M \cup S$, $M \cap S = \emptyset$ and $E = \{(s, m) \in S \times M, (m, s) \in M \times S \mid \text{"}sensor\ s\ sees\ marker\ m\text{"}\}$ where M denotes the set of all markers and S the set of all sensors. We define the relation "s can see m indirectly" as "there is a path in G from s to m". Here we outline proofs for the following two claims: (1) G is connected $\Leftrightarrow \forall s \in S\ \forall m \in M$: s can see m (indirectly) and (2) $\exists$ path p from $s \in S$ to $m \in M$ in G $\Rightarrow$ the position and orientation of sensor s can be expressed relative to marker m.

<u>Proof of claim 1 (by contradiction):</u>

(1.1) G is connected $\Rightarrow \forall s \in S\ \forall m \in M$: s can see m (indirectly)

Let G be connected, $s^* \in S$, $m^* \in M$ and s^* cannot see m^* (indirectly). Since s^* cannot see m^* indirectly, it follows from the definitions that there is no path in G from s^* to m^*. Since G is an undirected graph, it therefore follows that G is not connected which is a contradiction to the assumptions. Hence, the claim is true.

(1.2) G is connected $\Leftarrow \forall s \in S\ \forall m \in M$: s can see m (indirectly)

Let ∀sensor s ∀marker m : s can see m (indirectly). Assuming G is not connected, it follows by definition: $\exists s^* \in S, m^* \in M$: there is no path between s^* and m^*. Using the definition of the "sees-indirectly" relation it follows $\exists s^* \in S, m^* \in M$: $\overline{s^* \text{ sees } m^* \text{ indirectly}}$, which is a contradiction to the assumptions. Hence, the claim is true.

It follows: G is connected $\Leftrightarrow$ ∀sensor s ∀marker m : s can see m (indirectly).

<u>Calculations to express any sensor s in the coordinate frame any marker m (claim 2):</u>

$t_{m,s}$: position of marker m in sensor s' coordinate-frame

$R_{m,s}$: orientation of marker m in sensor s' coordinate-frame

$t_{m,n}$: position of marker m in marker n's coordinate-frame

$R_{m,n}$: orientation of marker m in marker n's coordinate-frame

Due to the calibration procedure we are given $t_{m,s}$ and $R_{m,s}$ for all sensor-marker-combinations for which the sensor s sees marker m, i.e. for which holds: $(s, m) \in E$.

Case 1: $(s, m) \in E$

The orientation of sensor s can be expressed in the marker m's coordinate-frame by $R_{s,m} = R_{m,s}^T$. Correspondingly, the position of sensor s can be expressed in marker m's coordinate-frame by $t_{s,m} = -R_{m,s}^T\, t_{m,s}$.

Case 2: $(s, m) \notin E$

Let $p = (s_1 = s, m_1, s_2, m_2, \dots, s_{N-1}, m_{N-1}, s_N, m_N = m)$ be a path from sensor s to marker m in G with $m_i \in M,\ s_i \in S$. Hence, $(s_i, m_i) \in E$ for $i = 1 \dots N$ and $(s_{i+1}, m_i) \in E$ for $i = 1 \dots N-1$. Due to the calibration procedure we are given t_{m_i,s_i}, $t_{m_i,s_{i+1}}$, R_{m_i,s_i} and $R_{m_i,s_{i+1}}$. Using the considerations for the first case we can easily calculate t_{s_i,m_i} and R_{s_i,m_i}. We are looking for $t_{s,m} = t_{s_1,m_N}$ and $R_{s,m} = R_{s_1,m_N}$.

1) Calculate for $i = 1 \dots N-1$:

$$t_{m_i,m_{i+1}} = R_{s_{i+1},m_{i+1}} t_{m_i,s_{i+1}} + t_{s_{i+1},m_{i+1}}$$

$$R_{m_i,m_{i+1}} = R_{s_{i+1},m_{i+1}} R_{m_i,s_{i+1}}$$

2) Calculate for $j = N-2 \dots 1$ (in that order):

$$R_{m_j,m_N} = R_{m_{j+1},m_N} R_{m_j,m_{j+1}}$$

$$t_{m_j,m_N} = R_{m_{j+1},m_N} t_{m_j,m_{j+1}} + t_{m_{j+1},m_N}$$

3) Calculate:

$$R_{s,m} = R_{s_1,m_N} = R_{m_1,m_N} R_{s_1,m_1}$$

$$t_{s,m} = t_{s_1,m_N} = R_{m_1,m_N} t_{s_1,m_1} + t_{m_1 m_N}$$
