# Supplementary material for: Validation of enhanced kinect sensor based motion capturing for gait assessment
Source: PLoS One. 2017 Apr 14;12(4):e0175813. doi: 10.1371/journal.pone.0175813 (PMC5391956; doi:10.1371/journal.pone.0175813)
Supplement: S2 Appendix — (PDF) [file pone.0175813.s002.pdf]

Joint position estimates are biased depending on the sensor's view angle

In order to evaluate how the view angle of the Kinect sensors might bias the joint position estimate, we have recorded a person while standing in the double support phase of a gait cycle (left foot in front of the right foot, at normal step width). Using two Kinect sensors, one from the left and one from the right, we recorded three-dimensional point clouds and skeletal information of the person. After reconstruction of the scene in the world coordinate system, we estimated a three-dimensional mesh of the body surface (Figure 5 in the main text) using the marching cubes algorithm in MeshLab (http://meshlab.sourceforge.net). Averaging the joint position estimates of both sensors, we gain a less biased estimate of the true joint location. For each joint we extract from the mesh the 400 closest vertices to the average joint position and determined by which sensor each vertex is visible while accounting for self-occlusion and the position and orientation of the sensors. Thereby we get two sets of surface vertices which are described by the visibility to the sensors. On the other hand, we have two joint position estimates, one from each sensor. Since the goal was to test whether the joint position estimates are closer to the surface which is visible to the respective sensor, we calculated for the four combinations the distances between the surface vertices and joint position estimate (see table below). We then determined the joints for which the median distance of the surface vertices to the joint estimate of the same sensor is significantly different than the median distance to the joint estimate of the other sensor (Wilkoxon signed test, A vs C and B vs C, see table below). Based on the results a contingency table has been filled (see table below). Fischer's exact reveals a non-random association between the closest joint estimate and surface ($p=0.03$).

Distances		Surface seen by sensor	
		left	right
Joint estimate of sensor	left	A	B
	right	C	D

	Wilcoxon's signed rank	
	A vs C	B vs D
SpineBase	**<0.001**	**<0.001**
SpineMid	**<0.001**	**<0.001**
Neck	**<0.001**	0.57
Head	**0.006**	**<0.001**

ShoulderLeft	**<0.001**	**<0.001**
ElbowLeft	0.08	0.74
WristLeft	**0.039**	0.78
HandLeft	0.38	0.08
ShoulderRight	**<0.001**	**<0.001**
ElbowRight	0.36	**0.004**
WristRight	**0.022**	**<0.001**
HandRight	0.07	**<0.001**
HipLeft	**<0.001**	**<0.001**
KneeLeft	0.15	0.20
AnkleLeft	**<0.001**	**<0.001**
FootLeft	**<0.001**	**<0.001**
HipRight	**0.025**	0.05
KneeRight	0.68	**<0.001**
AnkleRight	**<0.001**	**<0.001**
FootRight	**<0.001**	**0.026**
SpineShoulder	**<0.001**	0.59
HandTipLeft	**<0.001**	**<0.001**
ThumbLeft	0.76	0.15
HandTipRight	**<0.001**	**<0.001**
ThumbRight	**0.021**	0.56
**#joints with significantly different distance**	**18**	**16**

#joints		Surface seen by sensor	
		left	right
Joint estimate of sensor X is closest	left	9	2
	right	9	14
		18	16
