# Supplementary material for: Validation of enhanced kinect sensor based motion capturing for gait assessment
Source: PLoS One. 2017 Apr 14;12(4):e0175813. doi: 10.1371/journal.pone.0175813 (PMC5391956; doi:10.1371/journal.pone.0175813)
Supplement: S1 Fig — (PDF) [file pone.0175813.s003.pdf]

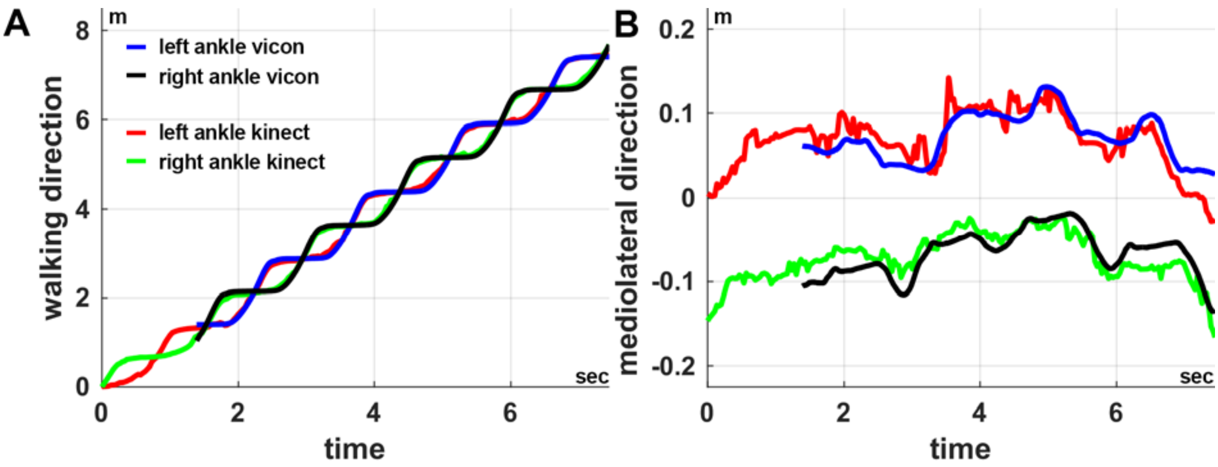

S1 Fig 1. Unfiltered ankle trajectories measured using the Kinect-based motion capturing system and VICON. In contrast to the data presented in the main text, the person here started walking outside the VICON tracking volume, which is about 6 meters long in our laboratory. (A) Ankle position in walking direction, (B) Ankle position in the mediolateral direction across time.
